# A high-sensitivity flexible bionic tentacle sensor for multidimensional force sensing and autonomous obstacle avoidance applications

**DOI:** 10.1038/s41378-024-00749-7

**Published:** 2024-10-21

**Authors:** Xinyu Liu, Kunru Li, Shuo Qian, Lixin Niu, Wei Chen, Hui Wu, Xiaoguang Song, Jie Zhang, Xiaoxue Bi, Junbin Yu, Xiaojuan Hou, Jian He, Xiujian Chou

**Affiliations:** 1 Science and Technology on Electronic Test and Measurement Laboratory, North University of China, 030051 Taiyuan, China; 2 School of Software, North University of China, 030051 Taiyuan, China

**Keywords:** Nanoscale materials, Carbon nanotubes and fullerenes

## Abstract

Bionic tentacle sensors are important in various fields, including obstacle avoidance, human‒machine interfaces, and soft robotics. However, most traditional tentacle sensors are based on rigid substrates, resulting in difficulty in detecting multidirectional forces originating from the external environment, which limits their application in complex environments. Herein, we proposed a high-sensitivity flexible bionic tentacle sensors (FBTSs). Specifically, the FBTS featured an ultrahigh sensitivity of 37.6 N^−1^ and an ultralow detection limit of 2.4 mN, which benefited from the design of a whisker-like signal amplifier and crossbeam architecture. Moreover, the FBTS exhibited favorable linearity (*R*^2^ = 0.98) and remarkable durability (more than 5000 cycles). This was determined according to the improvement in the uniformity of the sensing layer through a high-shear dispersion process. In addition, the FBTS could accurately distinguish the direction of external stimuli, resulting in the FBTS achieving roughness recognition, wind speed detection and autonomous obstacle avoidance. In particular, the ability of autonomous obstacle avoidance was suitably demonstrated by leading a bionic rat through a maze with the FBTS. Notably, the proposed FBTS could be widely applied in tactile sensing, orientation perception, and obstacle avoidance.

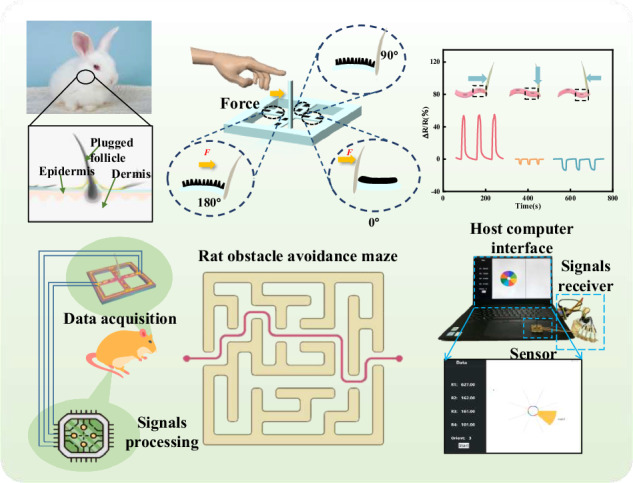

## Introduction

The ability of bionic tentacle sensors to sense slight pressure changes, especially within narrow surroundings, endows them with a crucial role in various fields, including obstacle avoidance^1,2^, human-machine interfaces^3–5^, and soft robotics^6–8^. The sensing mechanism of bionic tentacle sensors includes the piezoelectric effect^9–12^, piezoresistive effect^6,13,14^, capacitive effect^15,16^, and triboelectric effect^17^. Therefore, piezoresistive bionic tentacle sensors have attracted extensive interest because of their simple architectures, easy fabrication, limited environmental impacts, and simple back-end processing^18^. The sensing layer and device architecture are the two critical parts of a piezoresistive bionic tentacle sensor. Common sensitive materials include carbon nanomaterials (graphene^19^ and carbon nanotubes^20^), conductive polymers (polypyrrole^21^ and polythiophene^22^), and metal nanomaterials (Ag nanowires^23^, metal nanowires^24^, etc.). For example, Li et al. prepared a porous microstructured piezoresistive material comprising polydimethylsiloxane (PDMS) and multiwalled carbon nanotube (MWCNTs). Within the pressure range of 1 Pa to 1 kPa, the device exhibited the greatest linear response to the pressure^25^, with a sensitivity of 10.805 kPa^−1^. Zhu et al. proposed a piezoresistive sensor based on graphene-PDMS@sponge by fixing graphene onto a sponge skeleton using PDMS^26^. Piezoresistive sensors exhibit high elasticity (a strain up to 85%), high sensitivity (0.075 kPa^−1^), and a wide response range (0–50 kPa). Xia et al. described a piezoresistive sensor obtained by a self-assembled layer-by-layer technique from MXenes and carbon black (CB) on a polyurethane (PU) sponge^27^. The assembled sensor achieved reliable repeatability (more than 3700 cycles), high sensitivity (−5.26 kPa^−1^), and a wide sensing range (0–240 kPa). Notably, carbon nanotubes are susceptible to agglomeration due to notable van der Waals forces, and the novel two-dimensional multilayer conductive material (MXenes) facilitates the improvement in the sensitivity with a wider range of stresses, but the high cost of material synthesis limits its further application^28–30^. The above piezoresistive sensors exhibit a wide response range, whereas most sensors cannot detect the pressure at bionic tentacle sensors with high sensitivity and low detection limits that are suitable for large-scale preparation at a low cost are urgently needed.

In regard to sensing architectures, various architectures have been proposed. Chu et al. reported a novel two-dimensional piezoresistive sensor with a force resolution of nano-Newtons^31^. The sensor, which encompasses a silicon cantilever beam fabricated by monolithic micromachining technology can discriminate between laterally and vertically applied forces via clever electronic switches within a Wheatstone bridge circuit. Glass et al. introduced an interciliary contact sensing mechanism by 3D printing high-aspect-ratio polycaprolactone/graphene cilia structures, providing a sensor function similar to a switch for detecting air and water flow, braille detection, and debris recognition^32^. Ma et al. fabricated a novel micro-airflow sensor comprising four silicon nitride/silicon bent cantilever beams^33^. Yao et al. reported a capacitive 3D force‒tactile sensor based on a U-shaped slot structure that could identify the range of normal and tangential forces with high sensitivity and a fast dynamic response^34^. Guo et al. proposed a flexible bionic sensor based on the microstructure of an octopus sucker. The prepared octopus-based heuristic tactile sensor exhibited a high sensitivity of 0.636 kPa^−1^ and a wide linear sensing range (8–500 kPa)^16^. Most cantilever sensors operate along only a single direction and provide a single function, whereas a multifunctional tentacle sensor is needed in practical applications. The required functions include not only assessing the magnitude of forces but also recognizing the direction of forces^34,35^, surface texture nuances (e.g., roughness)^36^, airflow dynamics^37^, and other tactile sensations.

Herein, we proposed a novel flexible bionic tentacle sensor (FBTS) with high sensitivity, high precision, a low detection threshold and the ability to distinguish the stimulus direction. This sensor consists of a flexible cavity substrate, a flexible PI cross-beam film, MWCNT-sensitive layers, and nylon hairs. The MWCNTs were prepared via a high-shear dispersion process. The efficiency of force‒electric coupling in the sensing layer was improved because of the effective transfer of interfacial stress. Thus, the FBTS can detect low strains under flexible conformal conditions. The FBTS exhibits high sensitivity (37.6 N^−1^), favorable linearity (*R*^2^ = 0.98), and an ultralow detection limit of 2.4 mN. In addition, the as-prepared sensors functioned properly even after over 5,000 testing cycles, indicating their notable durability and repeatability. Furthermore, a bionic robot mouse successfully achieved autonomous obstacle avoidance when it was equipped with an FBTS, thereby integrating the design algorithm, a back-end resistance acquisition circuit, and an intelligent control and processing circuit.

## Materials and methods

### Preparation of MWCNT dispersion

MWCNTs and TNWDIS were purchased from Jiangsu XFNANO Co., Ltd., and Chengdu Organic Chemistry Co., Ltd., respectively. An MWCNT dispersion was prepared by a combination of physical mixing and noncovalent bonding surface modification processes. First, a small amount of surfactant TNWDIS was added dropwise to MWCNT powder, and this mixture was stirred for 30 min. Then, the obtained mixture was centrifuged for 5 min. Subsequently, the supernatant was collected, and a homogeneous and stable MWCNT dispersion was obtained.

### Preparation of flexible substrate

To achieve a suitable match and comfort for both human skin and animal skin under deformation, PDMS was chosen as the flexible polymer. The PDMS elastomer and curing agent (Sylgard 184) were purchased from Dow Corning. PDMS exhibits a low Young’s modulus, excellent flexibility, favorable tensile properties, and high corrosion resistance. In this work, two groups of PDMS mixtures (curing agent and crosslinking agent) were first prepared at a ratio of 10:1, followed by stirring at 500 r/min for 30 min. Then, air bubbles were removed under vacuum, and the mixture was poured into a square groove previously treated with a mold release agent. The square groove with the PDMS prepolymer was subsequently dried at 60 °C for 30 min to facilitate demolding. Finally, the bottom flexible liner was prepared. Subsequently, plasma treatment was performed to improve the hydrophilicity of the PDMS surface and to promote adhesion to the beam substrate^38^.

### Preparation of PI/MWCNT (PMC) film

First, PI beam electrode structures were fabricated. The polyimide (PI) film was split into beam structures. A 30-μm thick copper film was sputtered onto the PI substrate with magnetron sputtering equipment. The lead pattern was photolithographed by leveling, exposure, and development processes. Then, the film was solidified by heating and baking at 120 °C to form the metallic lead pattern, following wet etching for 20 s to remove the photoresist from the surface. After the metal leads had formed, the patterned metal lead substrate was placed in a press, and the press device was used at a high temperature and pressure to completely bond the cover film to the copper wires. Afterward, the PMMA layer and residual photoresist were removed with acetone. Next, the MWCNT solution was uniformly sprayed onto the beam substrate covered with the mask plate. Thereafter, the PI/MWCNT (PMC) film was placed on a heating table (LabTechEH20B, 70 °C) until the MWCNT dispersion was thoroughly dried. Finally, the PMC film was covered with a polyurethane film as an encapsulation layer.

### Assemble and glue the cilia

Nylon fibers with a diameter of 30 μm were obtained from a toothbrush (Darlie Deep Clean Series, Hao Lai Chemical Co., Ltd., China). The flexible PDMS substrate and PMC film were assembled. Next, 6–8 nylon fibers were inserted into the PI center, and UV-curing adhesive was applied at the center of the flexible sensitive structure and irradiated with a UV lamp for 5 min. As such, the FBTS was successfully fabricated. Finally, the electrodes exposed to the beam substrate were extracted and welded with silver paste.

### Characterization and measurement

The morphology of the as-prepared material was investigated by scanning electron microscopy (SEM; HITACHI-SU8010). The current was measured by a source meter (Keithley 2611B). During quantitative measurements, the FBTS was fixed to a linear motor. The tactile transducer was fixed on a sliding platform, and a digital force gauge (HANDPI, HP-2) was installed on another sliding platform to ensure that the force bar of the digital force gauge was vertically aligned with the bristle tip of the transducer. The resistance was measured in eight channels using a portable precision multichannel resistance tester (DAQ970A, China).

## Result and discussion

### Principle of the bioinspired ciliary sensor

**Fig. 1:**
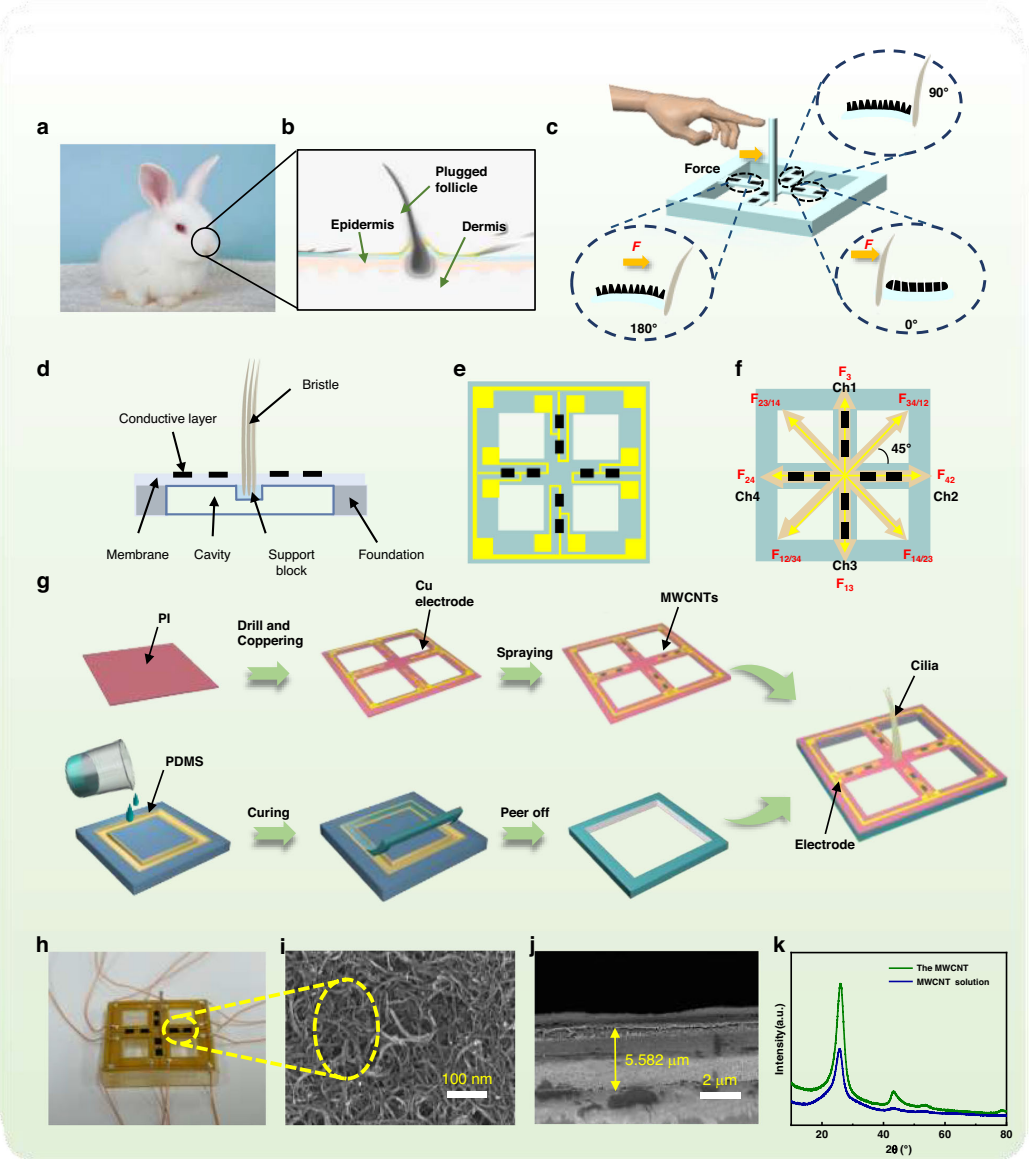
Working principle of the flexible whisker sensor based on a crossbeam structure. **a** Optical photograph and (**b**) schematic of a rabbit whisker sensory cell. **c** Schematic of the basic structure of the whisker sensor. **d** Detailed structure of a cross section of the sensor. **e** Electrode wiring diagram of the PMC layer of the sensor. **f** Top view of the sensor channels with different force directions. **g** Fabrication process of the whisker sensor. **h** Optical photograph of the as-prepared sensor. **i** SEM image of the MWCNT layer. **j** SEM image of the cross section of the MWCNT layer. **k** XRD patterns of the MWCNT dispersion and MWCNTs

Rabbit whiskers are very sensitive to mechanical signals. Specifically, when an external force is applied to a whisker, which deforms and stimulates the synapses of the nerve cells to generate a response, the mechanical signals are transformed into electrical signals in this process (Fig. 1a). Inspired by whiskers (Fig. 1b), a novel highly sensitive piezoresistive FBTS was proposed in this work, which can efficiently recognize the strength and direction of external forces. As shown in Fig. 1c, the proposed sensor comprises four key components: receptors made of whisker-like nylon fibers used to capture and amplify external stimuli, flexible crossbeams and two conductive layers on each crossbeam for signal perception, and an elastic substrate made of PDMS for conformal contact with human skin. Once the mechanical stimulus is captured by the receptors, it is immediately transmitted to the crossbeam at the bottom, resulting in deformation of the sensing material on the crossbeam, after which an electrical signal is generated. As larger deformation of the beam indicates higher sensitivity of the sensor, the cavity structure and the supporting layer were designed to improve the deformability of the crossbeam (Fig. 1d). The cavity supports a sufficiently large space for crossbeam deformation, while the support layer contributes to the tight bond between the receptor and crossbeam, which can reduce the energy loss during the transmission of mechanical signals. Moreover, this design can realize oblique homogeneous effects of the whisker-like receptor. The detailed conductive network mechanism of the sensor is shown in Figs. S1, S2.

Figure 1e shows a schematic of the PMC flexible film. The eight internal MWCNTs are connected to eight electrodes, and the four external electrode leads are connected to produce a closed loop. For the as-prepared sensor (Fig. 1f), when an external force is applied to channels 1 to 3, the sensitive materials in channel 1 are stretched, and the conductive networks R1 are separated from each other, leading to an increase in the resistance, whereas the sensitive materials in channel 3 are compressed, and the conductive networks become tighter, resulting in a decrease in the resistance. The shear forces F31 and F13 are opposite in direction, thereby generating opposite signal changes. Therefore, the direction of the shear force on the cilia can be determined by the resistance changes in the different channels. The force direction and corresponding state of the sensor are shown in Fig. S3. Obvious stress concentrations can be observed in microcracks within the sensitive layer.

Figure 1g shows the detailed fabrication process of the FBTS. The deformation map in Fig. S4 reveals the favorable flexibility of the sensor. The diameter of the MWCNTs is approximately 15 nm, and the thickness of the film is approximately 5 μm (Fig. 1j). The crystal structure of the MWCNT powder and dispersion were characterized via XRD (Fig. 1k). In addition, the elemental species and content of materials were analyzed via X-ray spectroscopy (EDS) (Fig. S5). The sharp diffraction peaks of the MWCNTs at nearly 26 °C reflect their high crystallinity, which contributes to the homogeneity and conductivity of the materials. Fig. S5a, b show layered EDS images of the MWCNT dispersion and the content of elements in the dispersion, respectively. C was the dominant element, accounting for 96.03% of the total dispersion, indicating the high purity of the as-prepared MWCNTs.

For the sensor, the cilia can be used as the cantilever beam of the sensor, and the stress in the beam can be expressed as

1 $$\sigma =\frac{32FL}{\pi {d}^{3}}$$

where *F* is the modulus of the mechanical stimulus, L is the distance between the locations of the stress and the bottom end of the cilia, and d is the diameter of the cilia. The strain can be expressed as

2 $$\varepsilon =\frac{\sigma }{E}$$

where *E* is the Young’s modulus of the cilia. With increasing external force, the strain at the root of the cilia increased. The simplified form of the normalized conductance of the bionic tentacle sensor can be expressed as^8^

3 $$S=\frac{1}{2}\left(1-erf\left(\frac{\mathrm{ln}\left(\frac{\varepsilon }{{\varepsilon }_{0}}\right)}{\mu }\right)\right)$$

where *erf (x)* is the error function, *ε* is the strain, *ε*_0_ and *μ* are the fitting parameters. The above equation shows the conductivity of the channel depends on the strain.

The alterable resistance of the sensor can be expressed as follows:

4 $${Rs}={Rs}1//{Rs}2\ldots //{Rsn}$$

5 $${Rc}={Rc}1//{Rc}2\ldots //{Rcn}$$

The total resistance (*R*_*T*_) of the sensor can be expressed as follows:

6 $${R}_{T}={R}_{1}+{R}_{2}+{R}_{s}+{R}_{c}$$

When a constant voltage is applied to the sensor, the sensitivity (*S*) of the sensor can be defined as

7 $$S=\frac{R-{R}_{0}}{\varDelta F}$$

where *R*_*0*_ is the initial resistance without external pressure and Δ*F* denotes the change of pressure.

**Fig. 2:**
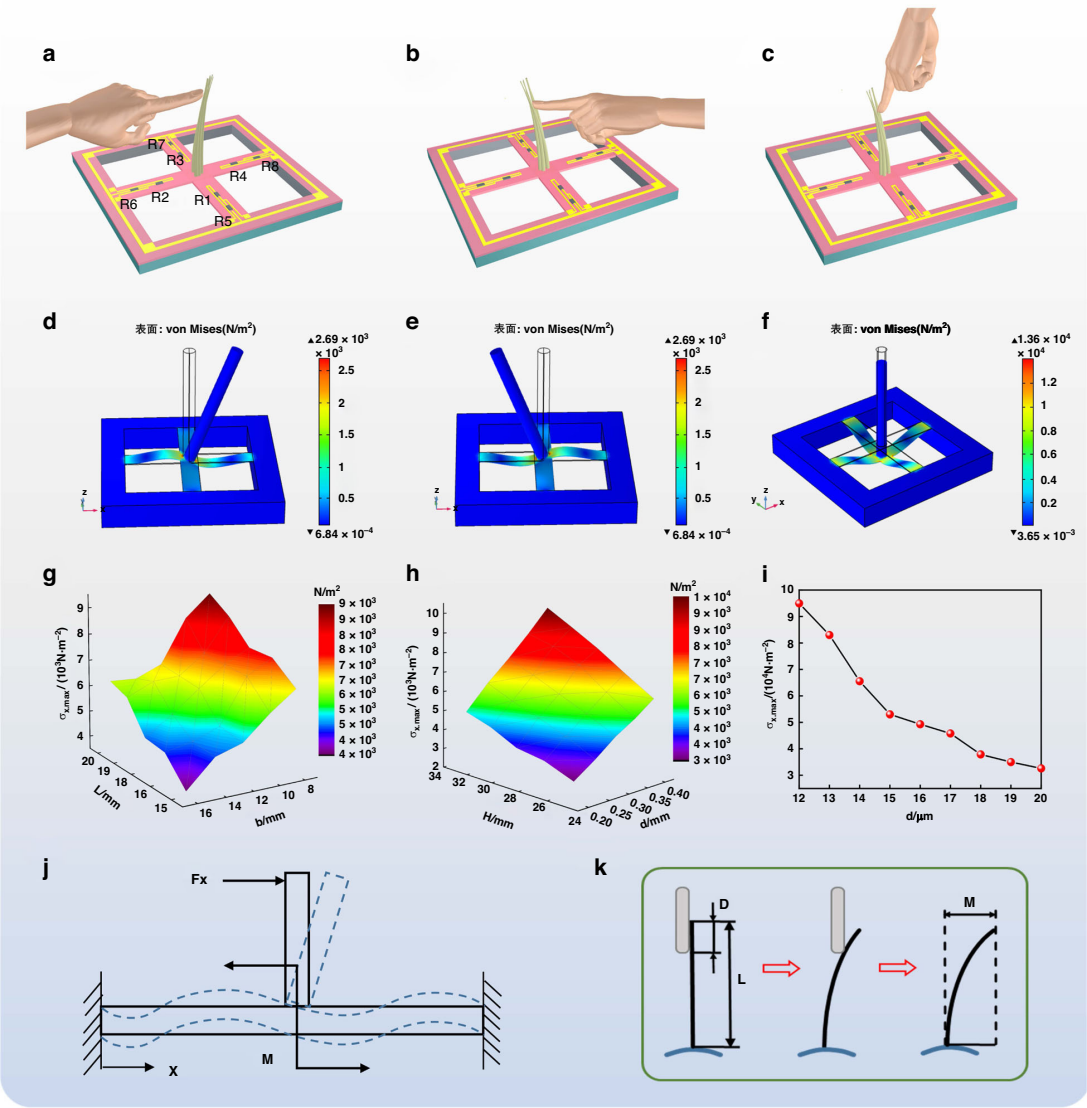
Simulation results for the multidimensional force of the FBTS. Schematic of different forces applied to the sensor: (**a**) Shear force of F24, (**b**) shear force of F42, and (**c**) normal force from top to bottom. **d**–**f** Simulation of the shear and normal forces applied to the sensor. **g** Relationship between the stress and beam length and width, (**h**) relationship between the stress and cilia length and diameter, (**i**) relationship between the stress and PI beam thickness. **j** Stress deformation diagram of the crossbeam of the sensor. **k** Diagram of the force applied to the cilia of the sensor

The sensing mechanism of the sensor was simulated via finite element analysis, as shown in Fig. 2a–c. The deformation process of the crossbeam was also recorded when an external force was applied to the sensor (Video S1). As shown in Fig. 2d, when the cilia are subjected to a shear force from the left, resistor R6 on the left side of the cilia is compressed, and R2 is stretched. The right resistor R4 is compressed, and R8 is stretched. As shown in Fig. 2e, when the cilia are subjected to an external shear force from the right, both R4 and R6 are stretched, and R2 and R8 are compressed. As shown in Fig. 2f, when the cilia are subjected to a top-down normal force, the stresses near the root and the outer border increase, indicating the ability of the sensor to recognize the direction of the external force. Figure 2g–i shows the variation in the stresses acting on the sensor with different component sizes. The results indicate that the maximum stress on the beams increases with increasing cantilever beam length, height of the cilia, and radius of the cilia, whereas it decreases with increasing width and thickness of the cantilever beam. According to the simulation results, the PMC membrane in this work is optimized as a square structure with a length of 3 cm and a thickness of 10 μm. The length and width of the four cantilever beams are set to 7 and 6 mm, respectively. The radius and length of the ciliary bundle are set to 1 and 15 mm.

A mechanical model of the FBTS is shown in Fig. 2j. When the force Fx is applied to the cilia, the horizontal component Fh along the x-axis and the vertical component M along the x-axis will be generated. Then, the two sensing materials on the left side of the beam are compressed and stretched, respectively, with the same force applied on the right side (from left to right). Therefore, the FBTS is more sensitive than are traditional beams with only a single sensing material on their structure. Figure 2k shows the distance D to touch the cilia and the distance M to bend the cilia. In subsequent tests, their effect on the signal output was determined.

### Output characterization

**Fig. 3:**
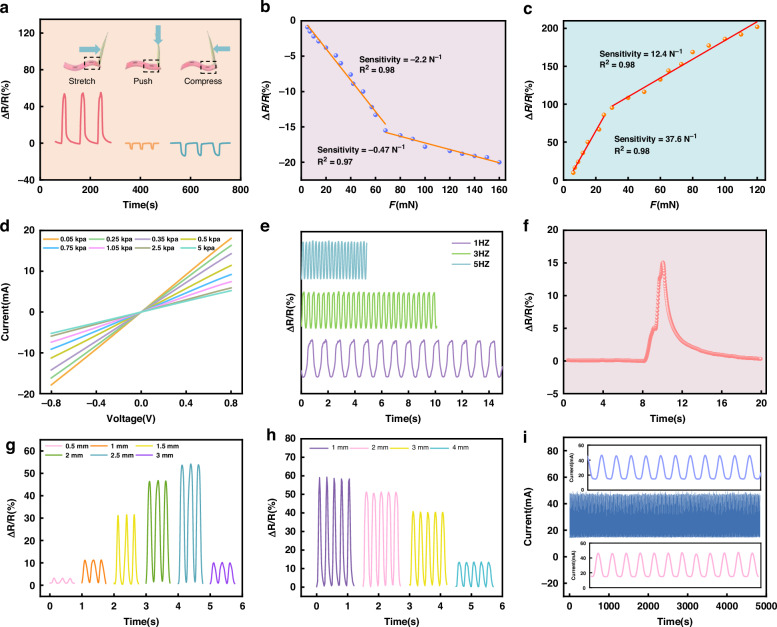
Sensing performance of the FBTS. **a** Signal changes in the cilia subjected to external forces along different directions. **b** Sensitivity of the cilia under a compressive shear force. **c** Sensitivity of the cilia under a tensile shear force. **d** *I*–*V* curves from −1 to 1 V under different pressures. **e** Signal output curves toward forces with different frequencies. **f** Response of the FBTS toward a shear force of 2.4 mN. **g** Response of the sensors under different bending distances M to the cilia. **h** Response of the sensors under different touching distances D to the cilia. **i** Stability of the FBTS toward a shear force of 50 mN for 5000 cycles

The resistance changes in the sensing layer were measured with an LCR instrument (2611B, Agilent). Figure 3a shows the resistance changes in the sensing layer when the cilia are subjected to stretching, compression, and downward pressure, respectively. The results demonstrated that the resistance of the sensing layer significantly increases when subjected to a stretch force, whereas the opposite trend occurs when subjected to compression and a downward force. Figure 3b shows the variation in resistance under a compressive shear force. After linear fitting, the relationship between the response and the external force can be divided into two linear parts: a low-pressure range (0-60 mN) with a high sensitivity of −2.2 N^−1^ and favorable linearity (R^2^ = 0.98), and a high-pressure range (60–160 mN) with a sensitivity of −0.47 N^−1^ and satisfactory linearity (R^2^ = 0.97). Figure 3c shows the change in resistance when the cilia are subjected to a stretching force. The resistance increases with increasing external force, showing a high sensitivity of 37.6 N^−1^ and an excellent linearity of 0.98 within the range of 0–25 mN, as well as a high sensitivity of 12.4 N^−1^ and a fine linearity of 0.98 within the range of 30–120 mN. The above results indicate that the as-prepared sensor can efficiently detect very low external forces, even at the μN level.

**Fig. 4:**
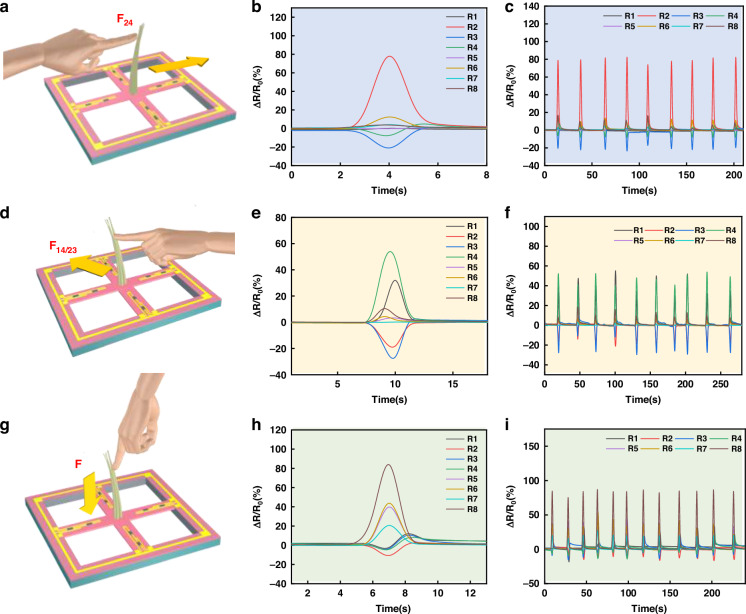
Sensing performance for the multichannel outputs of force sensors toward forces of different mechanical directions. Schematic of the forces applied to the sensors: (**a**) F24, (**d**) F14/23 shear force, and (**g**) downward force *F*. Eight-channel response toward (**b**) F24, (**e**) F14/23, and (**h**) downward force *F*. Loading-unloading multichannel cycles under (**c**) shear force F24, (**f**) F14/23, and (**i**) downward force *F*

As shown in Fig. 3d, all of the *I*–*V* curves of the tentacle sensor under different pressures exhibit favorable linear ohmic characteristics at a constant external voltage of 1 V. The slope of the *I*–*V* curve gradually decreases when the pressure increases from 0.05 to 5 kPa, indicating an increasing resistance of the sensor with increasing pressure. To investigate the stimulus-frequency dependence of the resistance, the samples were mounted between two fixtures on a customized uniaxial translational test rig for obtaining stimuli with different frequencies (Fig. S6). The results indicated a frequency-independent resistance of the sensor in the absence of a high frequency (Fig. 3e). Figure 3f shows the resistance change when the cilia are subjected to a shear force of 2.4 mN, illustrating the ultralow detection limit of the tentacle sensor. Furthermore, we investigated the effects of the cilia bending distance M and the cilia contact distance D on the sensing performance (Fig. 2k). The resistance first increases with bending distance M (0.5–2.5 mm) and then inversely decreases when M reaches 3 mm (Fig. 3g). However, the resistance continues to decrease with increasing cilia contact distance D (Fig. 3h). Moreover, the cyclic stability of the tentacle sensor was investigated by repeatedly loading and unloading the cilia with a force of 50 mN. The sensor signals remained relatively stable over 5000 cycles, indicating the high stability and durability of the sensor. The proposed sensor can also recognize the direction of the external force. This ability benefited from the different resistance changes in the different channels when the sensor was stimulated from a specific direction. The resistance changes in the different channels were recorded for demonstration by a DAQ970A + DAQM901A multichannel resistance tester when the sensors were stimulated from three distinct directions (Fig. 4). Specifically, when F24 was applied to the cilia, as shown in Fig. 4a–c, the conductive network was disconnected because of the tensile force acting on channel 2, resulting in a significantly increasing R2. When channel 4 was subjected to a compressive force, the resistance of R4 decreased, and the changes in R1, R3, and R5-R8 were not significant. When F14/23 was applied to the cilia, as shown in Fig. 4d–f, the conductive networks of channels 1 and 4 were disconnected due to the tensile force, leading to a significant increase in the resistance values of R1 and R4. When R2 and R3 were subjected to a compressive force, the conductive networks became more tightly connected, resulting in significant decreases in the resistance values of R2 and R3.

In addition, when a downward normal force is applied to the sensor, the external force is transmitted from the tip to the root of the cilia, and when the external components R5-R8 are subjected to a greater tensile force, a greater change in resistance of R5-R8 is observed. To investigate the response stability of the sensor to a stimulus from a specific direction; the responses were recorded when the sensor was repetitively stimulated (more than 200 times) from a specific direction. A constant result was obtained in this investigation, indicating satisfactory response stability to the stimulation direction of our proposed sensor (Fig. 4c, f, i). In addition, the responses of 8 sensor channels to stimulation from the other two directions are shown in Figs. S7 and S8, respectively. These results indicate that different responses can be generated in the 8 sensor channels to stimulations from different directions, while this response stability is also satisfactory. In this way, by analyzing the signal changes and amplitude magnitude for the 8 channels, the force direction can be suitably distinguished. Furthermore, the flexibility of the sensor was verified. As shown in Fig. S9, the dynamic resistance of the sensor was measured at bending angles of 0°, 30°, 45° and 60°. The sensor maintained an excellent sensing performance under bending. Fig. S10 shows the response of R1-R4 toward the loading and unloading of shear forces along 8 directions. Then, we summarize the sensing performance of recently reported tactile sensors in Table S1 and Note S2.

### Texture and gas flow detection based on tentacle sensor

**Fig. 5:**
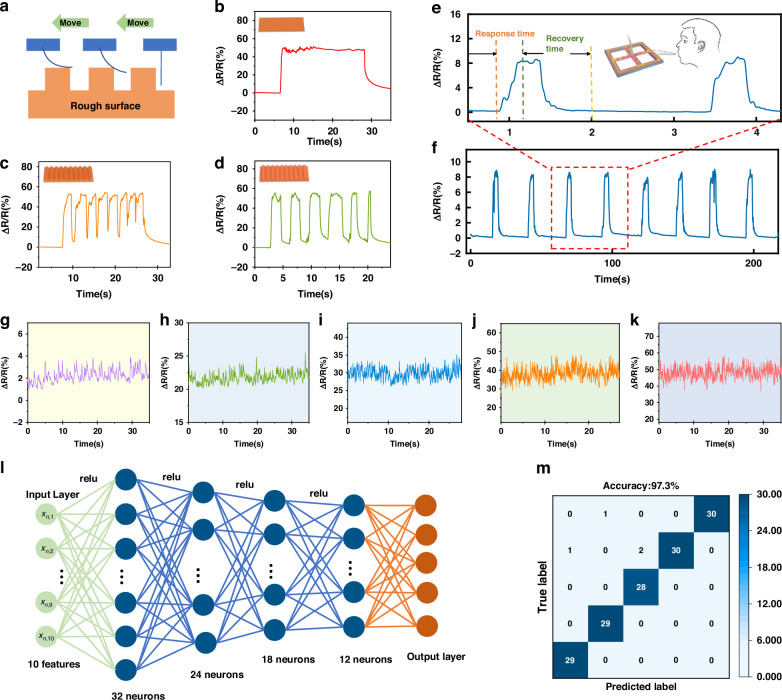
Sensing performance of the sensor toward texture roughness and gas flow. **a** Schematic of the surface texture. Sensor response to (**b**) a flat surface, (**c**) a surface with triangular grooves, and (**d**) a rectangular recessed surface; (**e**, **f**) sensor response to human blowing. **g**–**k** Sensor response to different wind speeds. **l** Schematic of an artificial neural network (ANN) for detection. **m** Confusion matrix based on 150 datasets of test data

In view of the excellent sensing performance of the proposed FBTS, it can likely recognize the surface texture of objects and gas flow^39^. To demonstrate texture recognition, a 3D printer was used to fabricate three plates with different surface textures: plate A is a smooth flat plate; there are 10 triangular grooves (1 cm in spacing and 5 mm in depth) on plate B; there are 10 rectangular grooves (5 mm in spacing and 5 mm in depth) on plate C. As shown in Fig. 5a, when the sensors can reach the surface of the object at a constant speed, the shape and number of grooves can be suitably recognized by recording the response signals of the FBTS.

In addition to their touch-sensing ability, sensors can detect noncontact forces. Figure 5e shows the response of the sensor toward blowing wind. The sensor exhibited a short response/recovery time of 0.23 s/0.53 s, indicating the real-time detection capability toward airflow. The sensor is then stimulated by blowing winds at different speeds. With increasing wind speed from 1 to 8 m/s, the RCR increases linearly, indicating an excellent recognition capability toward wind speeds (Fig. 5g–k). On this basis, a deep neural network-based model is proposed to parse and classify the signals obtained from the FBTS to recognize different airflow velocities. A total of 329 measured and dimensionless eigenvalues were extracted for each output signal and all the extracted features were then normalized. We constructed a model using a fully connected neural network consisting of an input layer, four hidden layers, and one output layer (Fig. 5l). The model was trained via 4588 sets of data, where a loss function based on cross-entropy and an Adam optimizer with a learning rate of 0.001 were used, with the batch size set to 64, for a total of 300 periods. On the basis of the classification results for 150 different sets of randomly selected samples, the mixing latent matrix was generated, as shown in Fig. 5m. The proposed neural network model achieved a high identification accuracy (97.3%) for wind speeds. The results verify the notable reliability of the machine learning framework and the FBTS, indicating the great potential of the proposed sensors for gas flow detection and turbulence detection.

### Bionic rat for obstacle avoidance based on FBTS

**Fig. 6:**
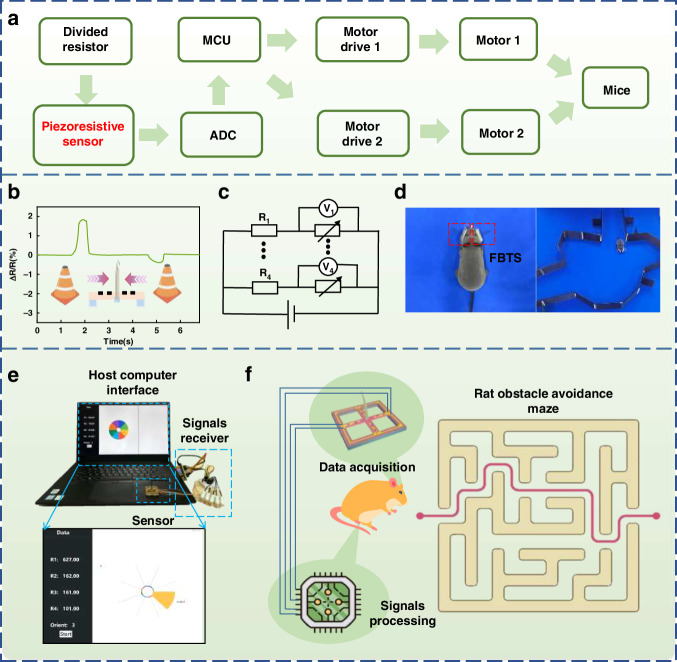
Bionic rat for obstacle avoidance based on FBTS and sensing data visualization. **a** Preparation procedures for the bionic obstacle avoidance rat. **b** Response of the tentacles toward forces along the opposite direction. **c** Piezoresistive signal acquisition principle diagram. **d** Bionic rat equipped with an FBTS and a simple maze. **e** Synchronous host computer interface diagram. **f** Diagram of autonomous obstacle avoidance of the bionic rat in the maze

Bionic rats possess great potential in space detection and autonomous obstacle avoidance. When a rat encounters an obstacle ahead, its tentacles sense changes in pressure, and obstacle avoidance is then realized. In this study, by attaching a cilia sensor to the head of a bionic rat, a bionic rat with the ability to avoid autonomous obstacles was successfully designed. As shown in Fig. 6a, the voltage across the resistors of each sensor channel was collected via the AD acquisition module. The MCU can internally calculate the resistance change in each channel according to Ohm’s law. When the bionic rat touches external obstacles while walking, the resistance change in each sensor channel differs for different stimulation directions. With the use of the MCU to decouple the resistance of the eight channels, the orientation of the obstacle can be obtained by the robot rat. Then, the obstacle avoidance instruction is generated, and the bionic rat is driven to avoid obstacles. A signal diagram of the forward and reverse forces applied to the cilia is shown in Fig. 6b. a circuit schematic diagram is shown in Fig. 6c. The controllable movement of bionic rats was achieved by adjusting the force applied to the sensor (Video S2). Photographs of a simple maze and a bionic rat equipped with an FBTS at the temples are shown in Fig. 6d. This bionic rat showed an excellent capability for autonomous obstacle avoidance and successfully traversed the maze (Video S3). Figure 6f shows the working mechanism and a diagram of bionic obstacles avoidance mouse in the maze. Finally, we performed simultaneous orientation recognition of the sensor within eight force quadrants. The interface of the host computer and a corresponding video are shown in Fig. 6e and S4, respectively.

## Conclusion

In summary, we reported an FBTS with ultrahigh sensitivity, an ultralow detection limit and a capability of force direction recognition. Specifically, an ultrahigh sensitivity of 37.6 N^−1^ and an ultralow detection limit of 2.4 mN were obtained for this FBTS. These excellent performance levels were obtained according to the design of a whisker-like signal amplifier and crossbeam architecture. The stability of our proposed sensor was verified by analyzing the FBTS repeatedly (more than 5000 times). In addition, the FBTS can accurately distinguish the direction of external stimuli. Applications of this recognition capability for roughness recognition, wind speed detection and autonomous obstacle recognition avoidance were demonstrated in this work. Notably, a bionic rat equipped with an FBTS successfully traversed a maze via the autonomous obstacle avoidance function. Therefore, our proposed FBTS shows great potential in tactile sensing, direction detection, and obstacle avoidance.

## Supplementary information

Supplymentary information

Video S1

Video S2

Video S3

Video S4

## References

[1] Xu, J., Xie, Z., Yue, H., Lu, Y. & Yang, F. A triboelectric multifunctional sensor based on the controlled buckling structure for motion monitoring and bionic tactile of soft robots. *Nano Energy* **104**, 107845 (2022).

[2] Xiong, W. et al. Multifunctional tactile feedbacks towards compliant robot manipulations via 3D-shaped electronic skin. *IEEE Sens. J.* **22**, 9046–9056 (2022).

[3] Xu, C. et al. Flexible pressure sensors in human-machine interface applications. *Small* **20**, 2306655 (2024). 10.1002/smll.202306655 38009791

[4] Liao, X. et al. A highly stretchable and deformation-insensitive bionic electronic exteroceptive neural sensor for human-machine interfaces. *Nano Energy* **80**, 105548 (2021).

[5] Gong, Y. et al. Wireless human-machine interface based on artificial bionic skin with damage reconfiguration and multisensing capabilities. *Acs Appl. Mater. Interfaces* **14**, 47300–47309 (2022). 36202397 10.1021/acsami.2c14907

[6] Wu, T. et al. Intelligent soft robotic fingers with multi-modality perception ability. *Iscience* **26**, 107249 (2023). 37502261 10.1016/j.isci.2023.107249 PMC10368832

[7] Tavakoli, M. et al. Autonomous selection of closing posture of a robotic hand through embodied soft matter capacitive sensors. *IEEE Sens. J.* **17**, 5669–5677 (2017).

[8] Jiao, Z. et al. Self-sensing actuators with programmable actuation performances for soft robots. *Sci. China-Technol. Sci.* **66**, 3070–3079 (2023).

[9] Motoo, K., Arai, F. & Fukuda, T. Piezoelectric vibration-type tactile sensor using elasticity and viscosity change of structure. *IEEE Sens. J.* **7**, 1044–1051 (2007).

[10] Ge, R., Yu, Q., Zhou, F., Liu, S. & Qin, Y. Dual-modal piezotronic transistor for highly sensitive vertical force sensing and lateral strain sensing. *Nat. Commun.* **14**, 6315 (2023). 37813847 10.1038/s41467-023-41983-3 PMC10562489

[11] Yu, Q. et al. Highly sensitive strain sensors based on piezotronic tunneling junction. *Nat. Commun.* **13**, 778 (2022). 35140219 10.1038/s41467-022-28443-0 PMC8828782

[12] Zhang, S. et al. Strain-controlled power devices as inspired by human reflex. *Nat. Commun.* **11**, 326 (2020). 31949147 10.1038/s41467-019-14234-7 PMC6965117

[13] Thanh-Vinh, N., Binh-Khiem, N., Takahashi, H., Matsumoto, K. & Shimoyama, I. High-sensitivity triaxial tactile sensor with elastic microstructures pressing on piezoresistive cantilevers. *Sens. Actuators A—Phys.* **215**, 167–175 (2014).

[14] Hua, Q. et al. Skin-inspired highly stretchable and conformable matrix networks for multifunctional sensing. *Nat. Commun.* **9**, 244 (2018). 29339793 10.1038/s41467-017-02685-9 PMC5770430

[15] Muhammad, H. B. et al. Development of a bioinspired MEMS based capacitive tactile sensor for a robotic finger. *Sens. Actuators A—Phys.* **165**, 221–229 (2011).

[16] Guo, X. et al. Highly sensitive and wide-rangeflexible bionic tactile sensors inspired by the octopus sucker structure. *Acs Appl. Nano Mater.* **5**, 11028–11036 (2022).

[17] Wang, J. J. et al. A stretchable self-powered triboelectric tactile sensor with EGaIn alloy electrode for ultra-low-pressure detection. *Nano Energy* **89**, 10.1016/j.nanoen.2021.106320 (2021).

[18] Duan, L., D'hooge, D. R. & Cardon, L. Recent progress on flexible and stretchable piezoresistive strain sensors: from design to application. *Prog. Mater. Sci.* **114**, 100617 (2020).

[19] Zhang, Z. et al. Plasmonic sensors based on graphene and graphene hybrid materials. *Nano Convergence* **9**, 28 (2022). 35695997 10.1186/s40580-022-00319-5 PMC9192873

[20] Schroeder, V., Savagatrup, S., He, M., Lin, S. & Swager, T. M. Carbon nanotube chemical sensors. *Chem. Rev.* **119**, 599–663 (2019). 30226055 10.1021/acs.chemrev.8b00340 PMC6399066

[21] Liang, X. et al. Split-half-tubular polypyrrole@sulfur@polypyrrole composite with a novel three-layer-3D structure as cathode for lithium/sulfur batteries. *Nano Energy* **11**, 587–599 (2015).

[22] Li, H. et al. Ultrahigh-sensitivity piezoresistive pressure sensors for detection of tiny pressure. *Acs Appl. Mater. Interfaces* **10**, 20826–20834 (2018). 29847907 10.1021/acsami.8b03639

[23] Cao, Z., Wang, R., He, T., Xu, F. & Sun, J. Interface-controlled conductive fibers for wearable strain sensors and stretchable conducting wires. *Acs Appl. Mater. Interfaces* **10**, 14087–14096 (2018). 29613767 10.1021/acsami.7b19699

[24] Tang, J. F., Fang, C. C. & Hsu, C. L. Enhanced organic gas sensor based on Cerium- and Au-doped ZnO nanowires via low temperature one-pot synthesis. *Appl. Surf. Sci.* **613**, 156094 (2023).

[25] Li, W. et al. Synergy of porous structure and microstructure in piezoresistive material for high-performance and flexible pressure sensors. *Acs Appl. Mater. Interfaces* **13**, 19211–19220 (2021). 33863232 10.1021/acsami.0c22938

[26] Jing, Z. et al. Highly sensitive, reliable and flexible piezoresistive pressure sensors based on graphene-PDMS @ sponge. *J. Micromech. Microeng.* **30**, 085012 (2020).

[27] Xia, H. et al. High sensitivity, wide range pressure sensor based on layer-by-layer self-assembled mxene/carbon black@polyurethane sponge for human motion monitoring and intelligent vehicle control. *IEEE Sens. J.* **22**, 21561–21568 (2022).

[28] Du, T. et al. MXene-based flexible sensors: materials, preparation, and applications. *Adv. Mater. Technol.* **8**10.1002/admt.202202029 (2023).

[29] Solangi, N. H., Mubarak, N. M., Karri, R. R., Mazari, S. A. & Jatoi, A. S. Advanced growth of 2D MXene for electrochemical sensors. *Environ. Res.* **222**, 115279 (2023). 36706895 10.1016/j.envres.2023.115279

[30] Chae, A. et al. Highly oxidation-resistant and self-healable mxene-based hydrogels for wearable strain sensor. *Adv. Funct. Mater.* **33**10.1002/adfm.202213382 (2023).

[31] Duc, T. C., Creemer, J. E. & Sarro, P. M. Piezoresistive cantilever beam for force sensing in two dimensions. *IEEE Sens. J.* **7**, 96–104 (2007).

[32] Glass, P. et al. 3D-printed artificial cilia arrays: a versatile tool for customizable mechanosensing. *Adv. Sci.* **10**, 10.1002/advs.202303164 (2023). 10.1002/advs.202303164 PMC10502633 37483144

[33] Ma, R. H. et al. A microcantilever-based gas flow sensor for flow rate and direction detection. *Microsyst. Technol.-Micro- Nanosyst.-Inf. Storage Process. Syst.* **15**, 1201–1205 (2009).

[34] Yao, T. et al. Highly sensitive capacitive flexible 3D-force tactile sensors for robotic grasping and manipulation. *J. Phys. D.—Appl. Phys.* **53**, 445109 (2020).

[35] Liu, Z. et al. 3D-structured stretchable strain sensors for out-of-plane force detection. *Adv. Mater.* **30**, 1707285 (2018). 10.1002/adma.201707285 29774617

[36] Cao, Y. et al. Fingerprint-inspired flexible tactile sensor for accurately discerning surface texture. *Small* **14**, 10.1002/smll.201703902 (2018). 10.1002/smll.201703902 29504238

[37] Asadnia, M. et al. From biological cilia to artificial flow sensors: biomimetic soft polymer nanosensors with high sensing performance. *Sci. Rep.* **6**, 32955 (2016). 27622466 10.1038/srep32955 PMC5020657

[38] Tony, A. et al. A preliminary experimental study of polydimethylsiloxane (PDMS)-to-PDMS bonding using oxygen plasma treatment incorporating isopropyl alcohol. *Polymers* **15**, 1006 (2023). 36850290 10.3390/polym15041006 PMC9958961

[39] Guo, X. et al. Highly stretchable, responsive flexible dual-mode magnetic strain sensor. *Adv. Mater. Technol.* **8**, 10.1002/admt.202201439 (2023).

