# Supplementary material for: A high-sensitivity flexible bionic tentacle sensor for multidimensional force sensing and autonomous obstacle avoidance applications
Source: Microsyst Nanoeng. 2024 Oct 21;10:149. doi: 10.1038/s41378-024-00749-7 (PMC11491448; doi:10.1038/s41378-024-00749-7)
Supplement: Supplementary file 1 — Supplymentary information [file 41378_2024_749_MOESM1_ESM.pdf]

Supplementary information

for

A High-Sensitivity Flexible Bionic Tentacle Sensor for Multi-Dimensional Force Sensing and Autonomous Obstacle Avoidance application

Xinyu Liu[1], Kunru Li[1], Shuo Qian[2], Lixin Niu[1], Wei Chen[1], Hui Wu[1], Xiaoguang Song[1], Jie Zhang[1], Xiaoxue Bi[1], Junbin Yu[1], Xiaojuan Hou[1], Jian He[1*], Xiujian Chou[1]

[1] *Science and Technology on Electronic Test and Measurement Laboratory, North University of China, Taiyuan 030051, China*

[2] *School of Software, North University of China, Taiyuan 030051, China*

*Corresponding author

E-mail address: drhejian@nuc.edu.cn

Supplementary Video Captions.

Video S1. The video recorded the whole process of simulating the stress of the sensor under lateral pressure and downward pressure direction force, showing that the stress is related to the cilia force.

Video S2. The video recorded the different reactions of the bionic mouse when blowing air into the sensor's cilia. When the cilia are blown from the back to the front, the bionic mouse moves forward, and when the cilia are blown from the front to the back, the bionic mouse moves backward, showing a high sensitivity of the cilia sensor.

Video S3. The video recorded the whole process of the mouse avoiding obstacles and navigating autonomously in the simple maze with the FBCTS fixed on the mouse's head, and finally finding the exit, showing good direction resolution and obstacle avoidance navigation ability.

Video S4. The video records the upper computer interface of synchronous recognition direction. When turn the cilia with fingers, the upper computer can synchronously display it. It shows that the sensor has good direction recognition performance.

Supplementary Note S1:

For the sensing mechanism of the sensor, as shown in Fig. S1(a), the total resistance consists of the variable resistance Rs of the MWCNTs, the contact resistance Rc between the MWCNTs and the electrodes, and the resistances R1 and R2 of the electrodes. In the state of low tensile or compression, the resistance Rc changes in the MWCNTs and the electrodes' direct contact. As the tensile or compressive force gradually increases, the change in the variable resistance Rs of the MWCNT dominates. As shown in Fig. S1(b), the conductive path of MWCNTs is continuously compressed with the increase of the external force, increasing to a maximum constant current value.

The structural changes of the conductive network inside the MWCNTs are reflected in S2. The green color indicates carbon nanotubes in the conductive pathway, and the red color indicates carbon nanotubes, which have good connectivity between adjacent MWCNTs. With the increase of external pressure, the tensile deformation causes a small movement of carbon nanotubes, and the conductive pathway is connected. If two carbon nanotubes are not in direct contact, but the mutual distance is less than the range of tunneling effect, the two form a carbon nanotube junction resistance R_j. The tunneling resistance between two SWCNTs is:

$$R_j = \frac{V}{AJ} = \frac{h^2 d}{Ae^2\sqrt{2m\lambda}} \exp\left(\frac{4\pi d}{h}\sqrt{2m\lambda}\right) \tag{8}$$

where J is the tunneling current density, V is the potential difference, e is the single-electron charge, m is the electron mass, h is Planck's constant, λ is the energy barrier height, and A is the cross-sectional area of the tunneling surface.

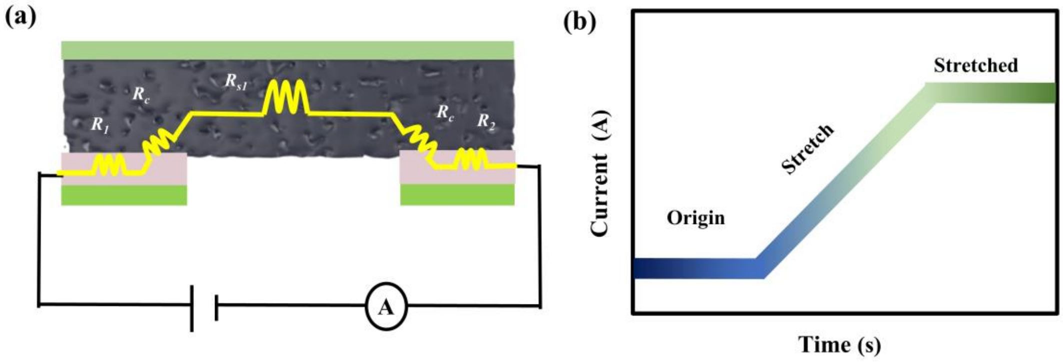

Fig. S1. (a) Sensing mechanism based on carbon nanotube conductive network pressure sensor. (b) Schematic of the current change of piezoresistive sensor.

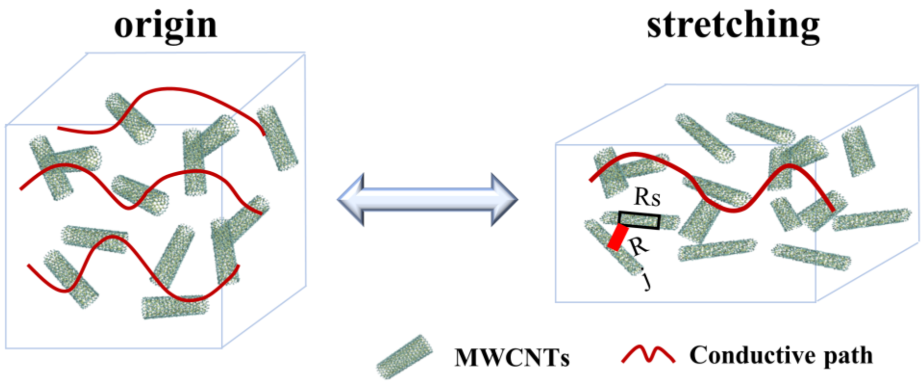

Fig. S2. Schematic diagram of the conductive network of the sensitive material, (red color indicates the conductive paths).

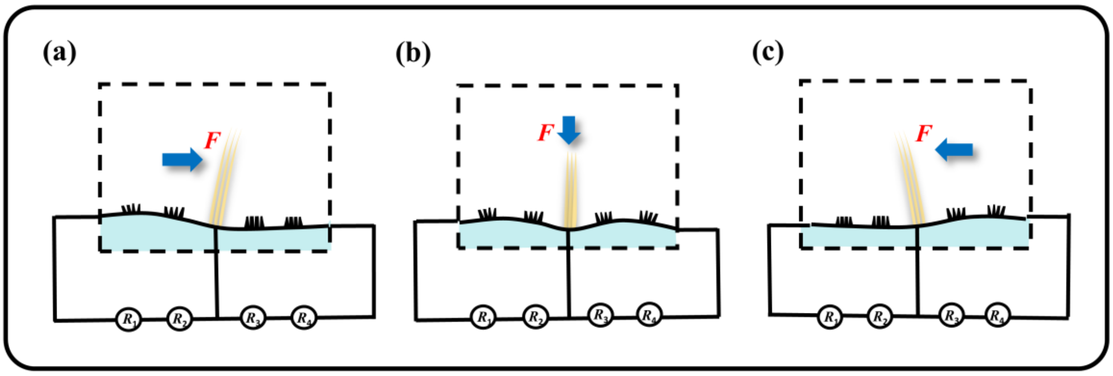

Fig. S3. Schematic diagrams showing the structure variations when a shear force is applied from left to right (a), from right to left (c), and a normal force (b) is applied from top to bottom.

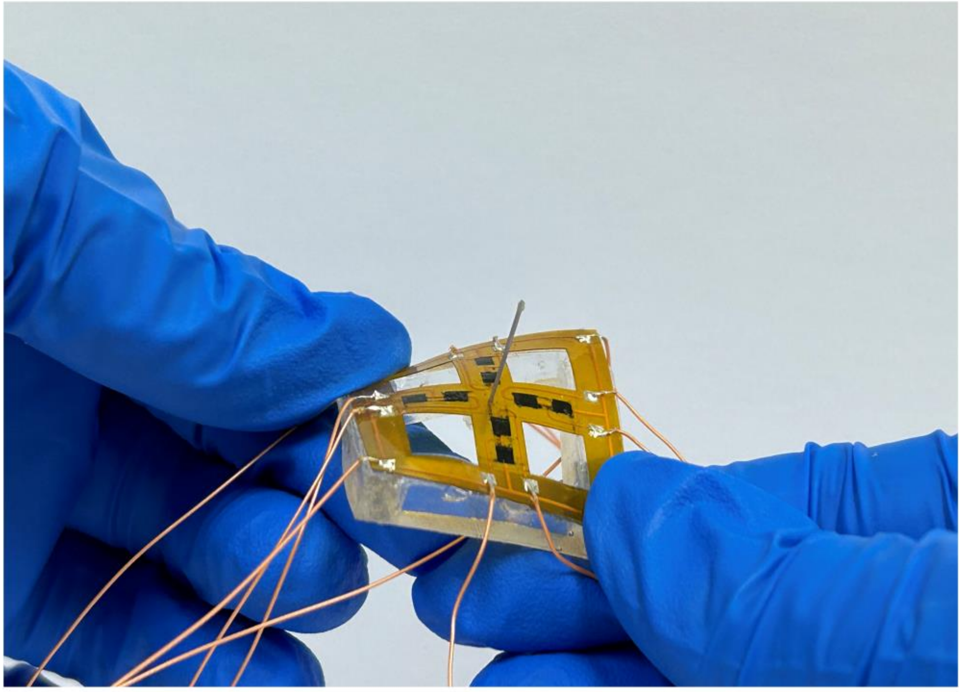

Fig. S4. The sensor deformation diagram, reflecting the good flexibility of the sensor.

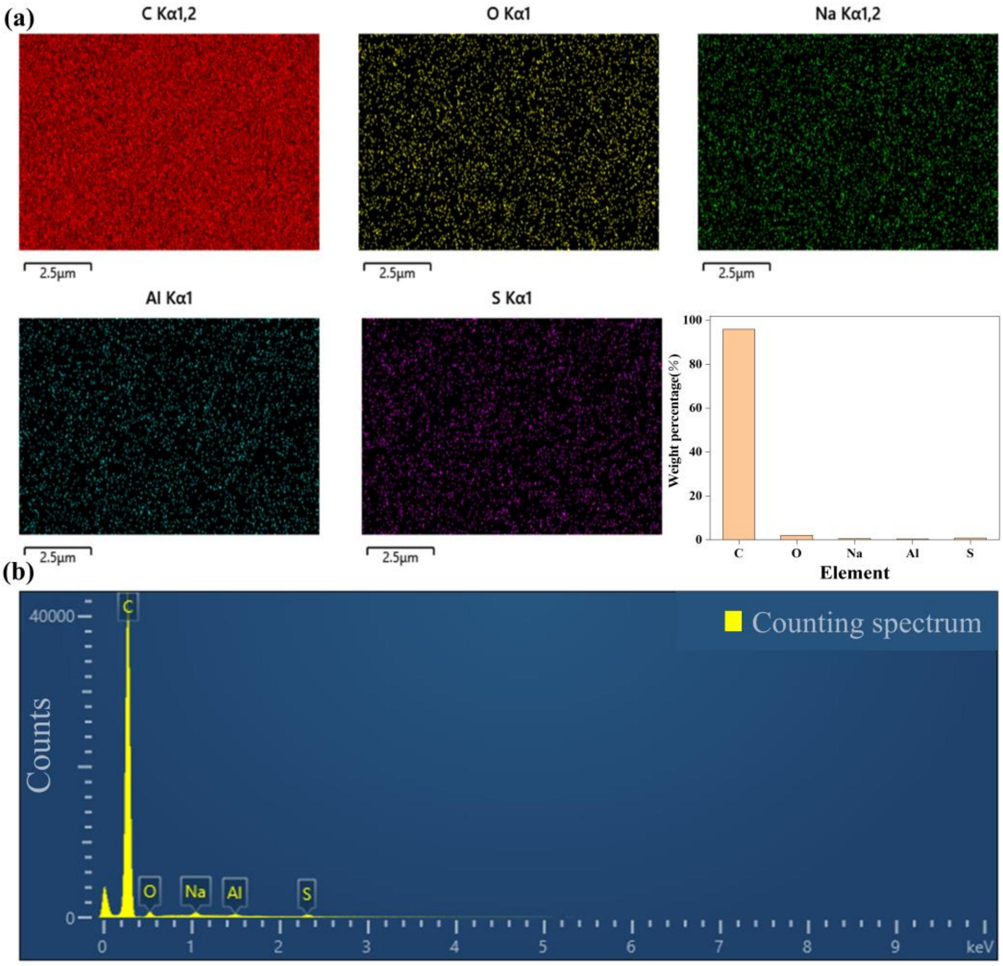

Fig. S5. X-ray spectroscopy (EDS) is used to analyze the compositional elemental species and content of materials in the microregion. (a) EDS spectra of carbon nanotubes. The EDS results show that the elemental contents of C, O, Na, Al, and S in the MWCNTs are 96.3wt%, 2.13wt%, 0.59wt%, 0.39wt%, and 0.86wt% respectively. Carbon is the main element. In addition, the atomic contents of O, Na, Al, and S constitute only 3.97wt % of the total material, which is normal for carbon nanotube dispersions. (b) The total number of carbon nanotube element distribution maps spectra.

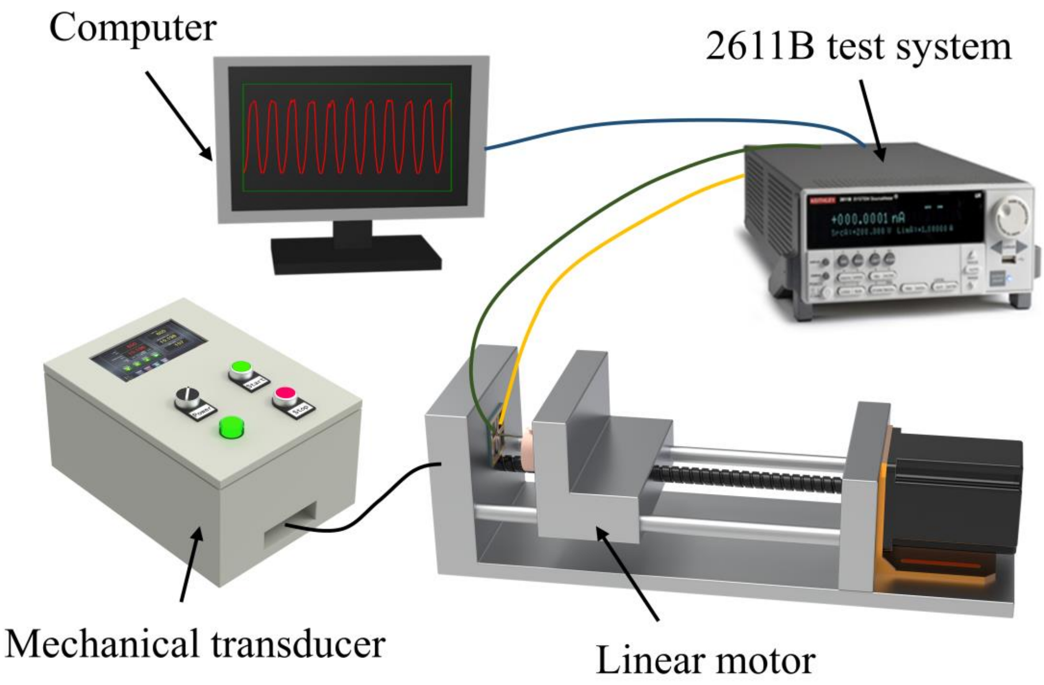

Fig. S6. The schematic diagram shows the measuring device of the tentacle sensor, in which the tentacle sensor is fixed to a linear motor for measurement.

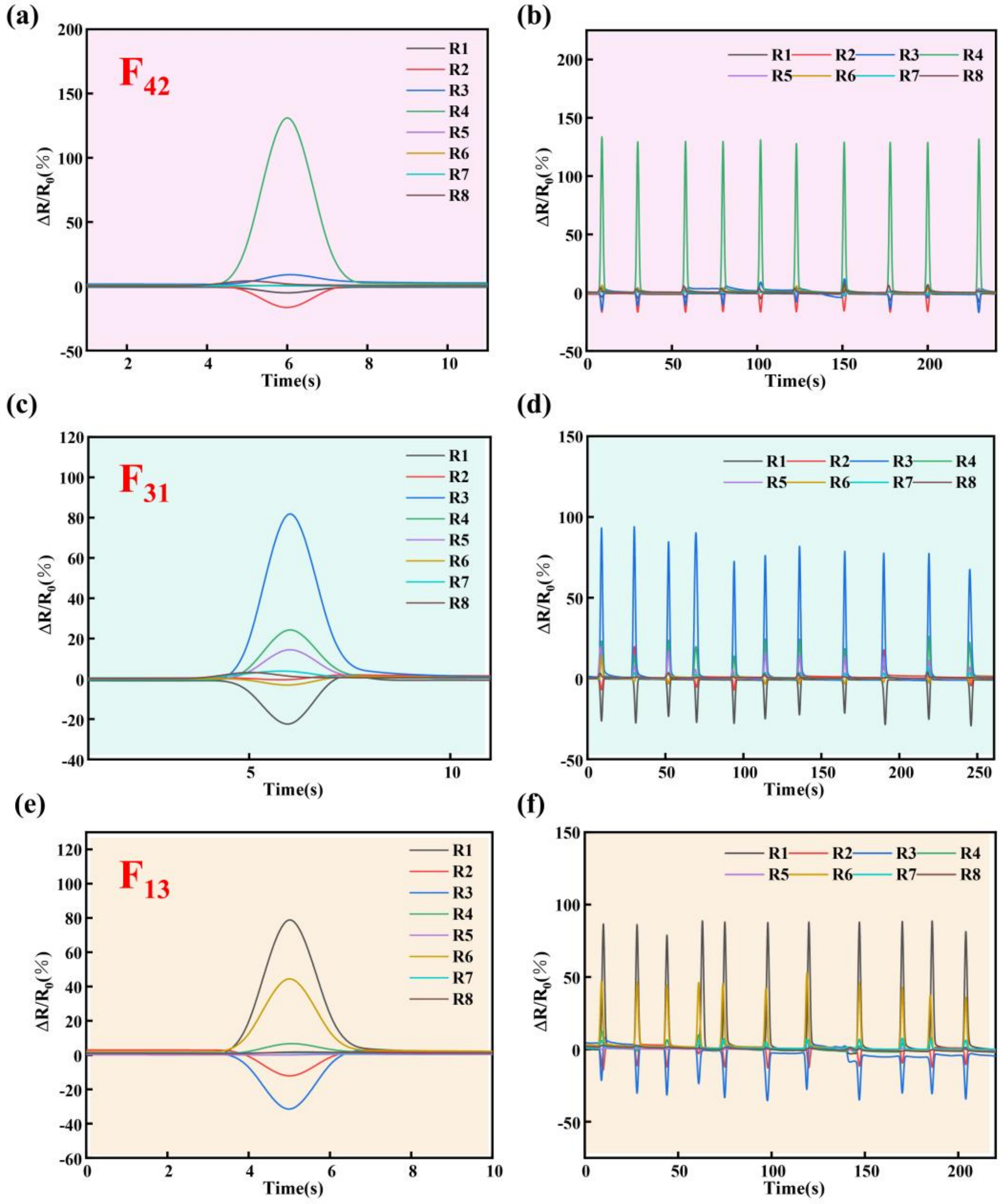

Fig. S7. (a, c, e) Eight-channel response behavior of F42, F31, and F13. (b,d,f) Multi-channel cyclic output of loading and unloading under shear forces F42, F31 and F13.

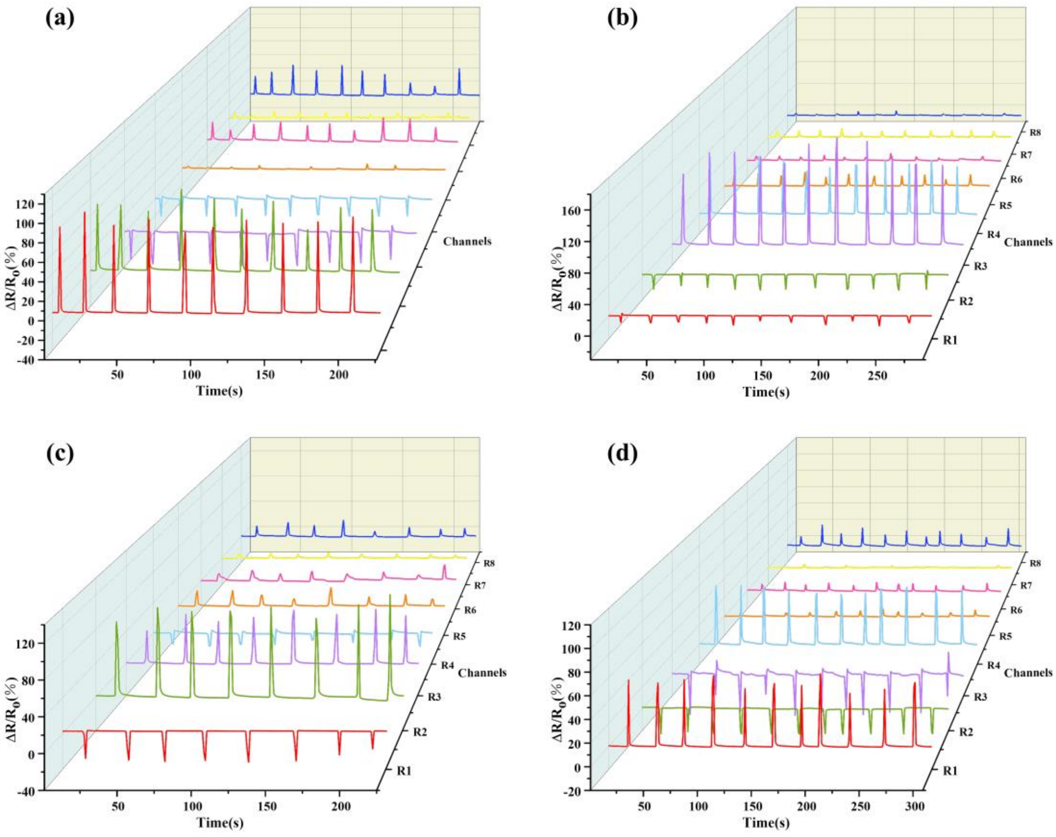

Fig. S8. The eight resistance response behaviors under different shear forces of F12/34, F34/12, F23/14 and F14/23.

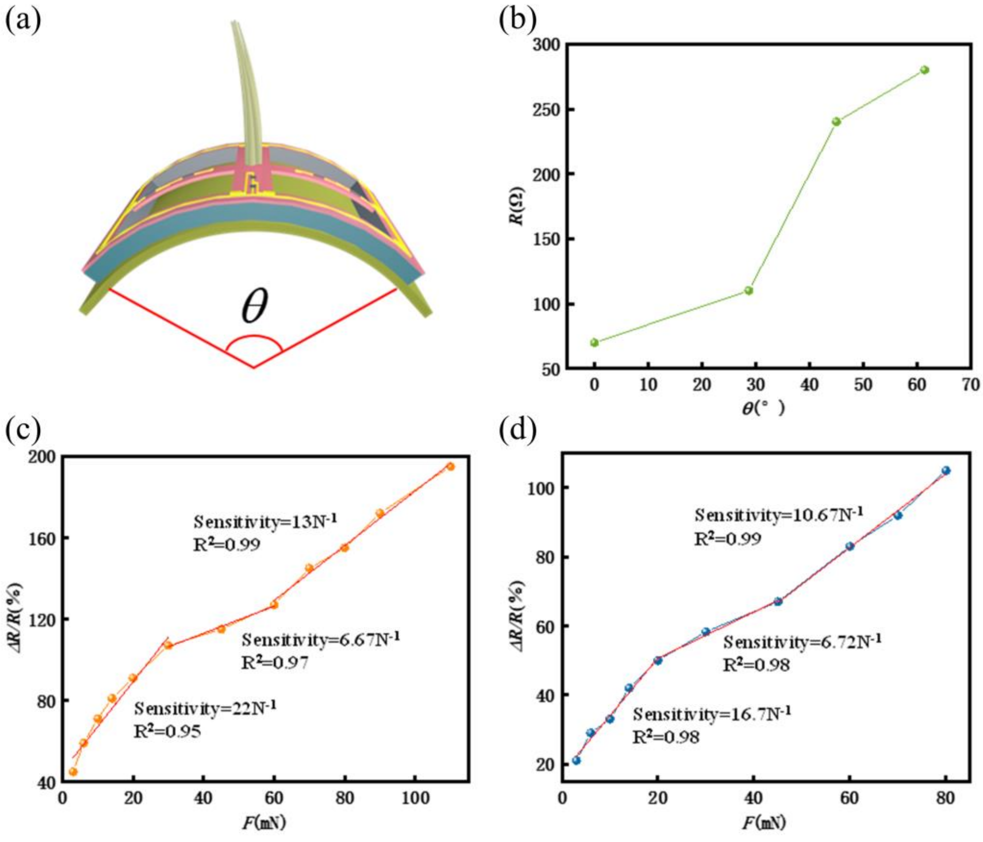

Fig. S9. The resistance change and sensitivity of the sensor under different bending angles.

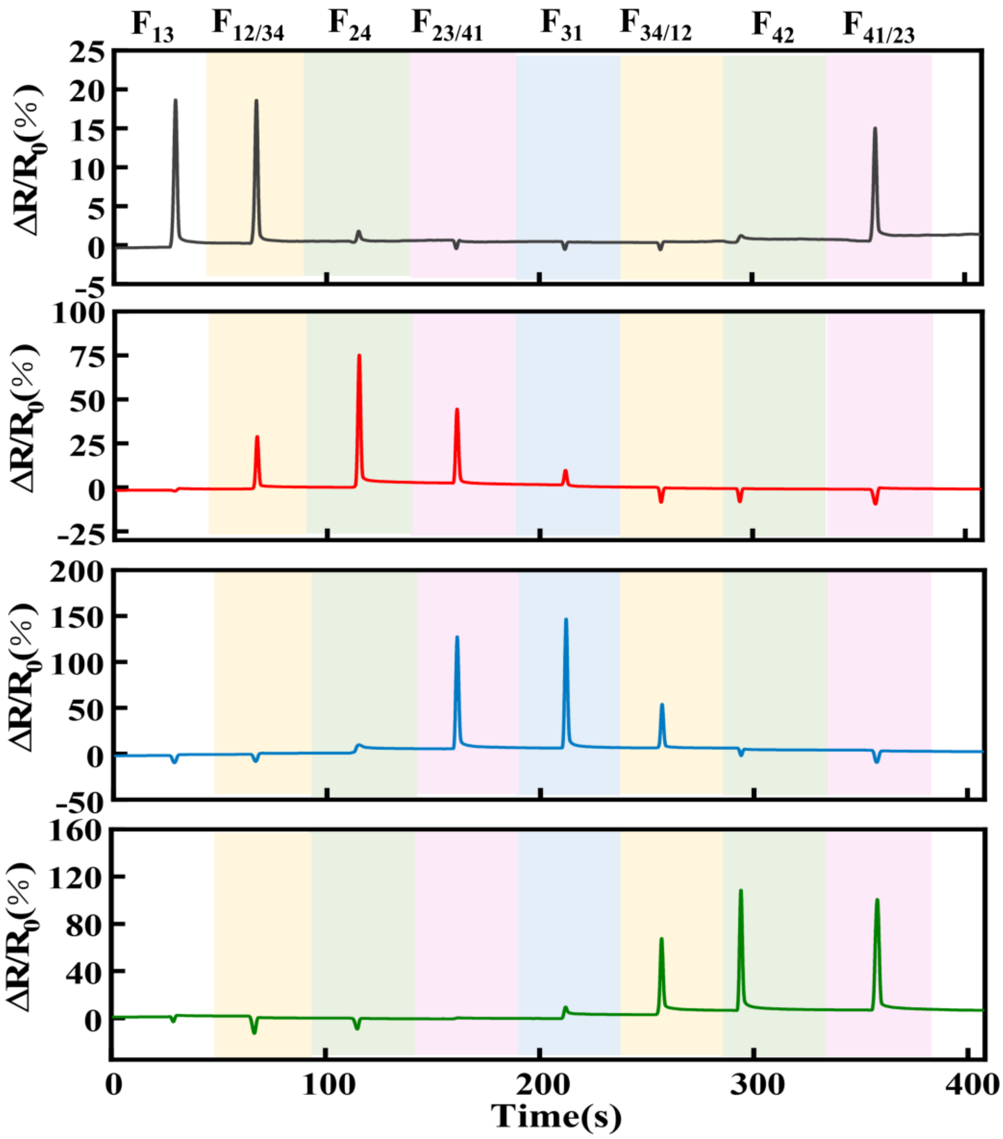

Fig. S10. R1-R4 resistors respond to shear forces in loading and unloading in eight different directions.

Table S1. Performance comparison of different pressure sensors in sensitivity, detection limit, and directional characteristics.

Conducting filler	Structure design	Sensitivity	Dectection limit	Sensing mechanism	Force direction dectection	Refs
Carbon	Carbonized paper	5.67kpa^{-1}	None	Piezoresistive	No	1
rGO	Pyramid structure	-5.53kPa^{-1}	None	Piezoresistive	No	2
MXene/SiNPs	Cotton fibers	12.23kPa^{-1}	None	Piezoresistive	No	3
MXene/rGO	Aerogels	0.28kPa^{-1}	60Pa	Piezoresistive	No	4
FEP、Acrylic and Copper	Double electrode	None	1.129μN	Triboelectric	No	5
Nylon/Carbon	Hair sensor	0.72N^{-1}	1mN	Piezoresistive	No	6
EGaIn/silicone	Fingerprint-like	None	16.88mN	Triboelectric	No	7
MWCNTs/PI	Cross-beam structure	37.6N^{-1}	2.4mN	Piezoresistive	Yes	This work

Supplementary Note S2:

As shown in Figure S1, we compared the directional resolution and mechanical sensitivity and detection limits of the sensor in this study with other reported bionic sensors. The bionic tentacle sensor based on cross beam presented in this paper has high sensitivity to the detection of forces in different directions. In addition, Table S1 compares the structural design and sensing mechanism of our proposed sensor with other reported sensors. The sensitivity of the above sensors is almost not as high as that of this paper. In this work, the sensor has a high mechanical sensitivity (37.6N^{-1}) and a low detection limit (2.4mN). In addition, the preparation process proposed in this paper is simple, cost-effective, and has obvious advantages. These sensors cannot have both of these properties. Therefore, compared with various existing tactile sensors, bionic tentacle sensors based on cross beams have unique advantages in terms of sensitivity and direction detection. These results show that the proposed tactile sensor has great application potential in robotics and bionic application scenarios.

References

1 Chen, S., Song, Y. J. & Xu, F. Flexible and Highly Sensitive Resistive Pressure Sensor Based on Carbonized Crepe Paper with Corrugated Structure. Acs Applied Materials & Interfaces 10, 34646-34654, doi:10.1021/acsami.8b13535 (2018).

2 Zhu, B. W. et al. Microstructured Graphene Arrays for Highly Sensitive Flexible Tactile Sensors. Small 10, 3625-3631, doi:10.1002/smll.201401207 (2014).

3 Wang, S. et al. Hierarchical design of waterproof, highly sensitive, and wearable sensing electronics based on MXene-reinforced durable cotton fabrics. Chemical Engineering Journal 408, doi:10.1016/j.cej.2020.127363 (2021).

4 Jiang, D. G. et al. Superelastic $Ti_3C_2T_x$ MXene-Based Hybrid Aerogels for Compression-Resilient Devices. Acs Nano 15, 5000-5010, doi:10.1021/acsnano.0c09959 (2021).

5 An, J. et al. Biomimetic Hairy Whiskers for Robotic Skin Tactility. Advanced Materials 33, doi:10.1002/adma.202101891 (2021).

6 Liu, Y. F. et al. A biomimetic multifunctional electronic hair sensor. Journal of Materials Chemistry A 7, 1889-1896, doi:10.1039/c8ta10750e (2019).

7 Qu, X. C. et al. Fingerprint-shaped triboelectric tactile sensor. Nano Energy 98, doi:10.1016/j.nanoen.2022.107324 (2022).
